# Immediate effects of an anchor system on the stability limit of individuals with chronic dizziness of peripheral vestibular origin^☆^

**DOI:** 10.1016/j.bjorl.2015.12.008

**Published:** 2016-03-29

**Authors:** Almir Resende Coelho, Ana Paula do Rego Andre, Júlia Licursi Lambertti Perobelli, Lilian Shizuka Sonobe, Daniela Cristina Carvalho de Abreu

**Affiliations:** a Universidade de São Paulo (FMRP-USP), Faculdade de Medicina de Ribeirão Preto, Programa em Reabilitação e Desempenho Funcional, Ribeirão Preto, SP, Brazil; b Universidade de São Paulo (FMRP-USP), Faculdade de Medicina de Ribeirão Preto, Ribeirão Preto, SP, Brazil

**Keywords:** Postural balance, Vestibular diseases, Dizziness, Haptic information, Equilíbrio postural, Doenças vestibulares, Tontura, Informação háptica

## Abstract

**Introduction:**

The symptoms associated with chronic peripheral vestibulopathy exert a negative impact on the independence and quality of life of these individuals, and many individuals continue to suffer from these symptoms even after conventional vestibular rehabilitation.

**Objective:**

To evaluate the acute effect of an anchor system for balance evaluation of patients with chronic dizziness who failed to respond to traditional vestibular rehabilitation.

**Methods:**

Subjects over 50 years of age, presenting with chronic dizziness and postural instability of peripheral vestibular origin, participated in the study. The limit of stability was evaluated in three positions using the Balance Master^®^ system: Position 1, standing with the arms along the body; Position 2, standing with the elbows bent at 90° (simulating holding the anchors); and Position 3, with the elbows bent at 90° holding the anchors. The variables of movement latency, endpoint excursion and directional control of movement were evaluated.

**Results:**

Using the anchor system, significant reduction of time in the response at the beginning of the movement compared to Position 1 (*p* < 0.05); increased endpoint excursion in the left lateral direction compared to Position 1 (*p* < 0.05); and more directional control of movement in the anterior and posterior directions (*p* < 0.05) compared to the other positions, were found.

**Conclusion:**

While using the system anchor, individuals with chronic peripheral vestibulopathy showed an immediate improvement in the stability limit in relation to the movement latency, endpoint excursion, and directional control of movement variables, suggesting that the haptic information aids postural control.

## Resumo

**Introdução:**

Os sintomas associados à vestibulopatia periférica crônica têm impacto negativo na independência e qualidade de vida dos indivíduos, e muitos deles continuam a sofrer desses sintomas, mesmo depois de terem passado pela reabilitação vestibular convencional.

**Objetivo:**

Avaliar o efeito agudo de um sistema de ancoragem para avaliação do equilíbrio de pacientes com tontura crônica que não responderam à reabilitação vestibular tradicional.

**Método:**

Indivíduos com mais de 50 anos que se apresentaram com tontura crônica e instabilidade postural de origem vestibular periférica participaram no estudo. O limite de estabilidade foi avaliado em três posições, com o uso do sistema Balance Master^®^: Posição 1, de pé com os braços pendentes ao longo do corpo; Posição 2, de pé com os cotovelos flexionados em 90° (simulando a posição de segurar as âncoras); e Posição 3, com os cotovelos flexionados em 90° e segurando as âncoras. Foram avaliadas as variáveis de latência de movimento, ponto final da excursão e controle direcional do movimento.

**Resultados:**

Com o uso do sistema de âncoras, ocorreu redução significante no tempo de resposta no início do movimento em comparação com a Posição 1 (*p* < 0,05); aumento no ponto final da excursão na direção lateral esquerda, em comparação com a Posição 1 (*p* < 0,05); e mais controle direcional do movimento nas direções anterior e posterior (*p* < 0,05), em comparação com as demais posições.

**Conclusão:**

Enquanto usavam o sistema de âncoras, os indivíduos com vestibulopatia periférica demonstraram melhora imediata no limite da estabilidade em relação às variáveis latência de movimento, ponto final da excursão e controle direcional do movimento, sugerindo que a informação háptica auxilia no controle postural.

## Introduction

The majority of symptoms reported by elderly people, such as dizziness, postural instability, gait disturbances and falling incidents,^1^ can be the consequences of several diseases originating in the vestibular system.^2^ These vestibular system disorders are a significant problem in healthcare.

Dizziness is prevalent in 5–10% of the world's population, and it is the seventh most frequent complaint in women and the fourth most frequent in men. Approximately 47% of men and 61% of women over age 70 years are affected by dizziness. Dizziness occurs in 65% of subjects aged 65 years and older who live in the community and in 81–91% of those of the same age who are treated at geriatric outpatient clinics.^3^

The deterioration of vestibular function lead to falling and has many consequences, including physical impairments (tissue injuries, gait changes); psychological changes (fear of falling, depression); social changes (isolation, dependency); and economic changes (medication costs, rehabilitation).

Postural control, a complex ability that involves postural orientation and the maintenance of balance, depends on central processing inputs related to visual, vestibular, and somatosensory afferent mechanisms,^4^ and to the proportional neuromuscular action of efferent mechanisms. The information related to visual, vestibular, and somatosensory systems should be integrated and selected in accordance with the environment and the type of task to be performed in order to maintain postural stability.^5^

In patients with vestibular disorders, disturbances of sensorial integration or information processing that generate conflict among the visual, vestibular and somatosensory systems may be present, which could explain the permanency of symptoms of chronic vestibular disorder, such as the inability to modulate sensory information in a way that is adequate to ensure postural balance.^2^

Among the main therapies recommended for the treatment of vestibular disorders, as well as for multiple otoneurologic symptoms, drug therapies, surgery, and vestibular rehabilitation (VR) should be highlighted. VR is a type of therapy characterized by its physiological action on the vestibular system, which acts on the central mechanisms of neuroplasticity to generate adaptation, habituation, or replacement mechanisms in the vestibular system and to relieve vestibular symptoms.^6^

It is believed that VR can promote healing in 30% of cases and can achieve other positive results in 85% of individuals.^7^ Therefore, it is important to search for effective ways to improve otoneurologic symptoms and to prevent falls, as well as for clinically viable assessments that can be introduced in the daily clinical practice for this population.

Mauerberg-Decastro^8^ developed an instrument aimed at improving corporal stability called the anchor system, which works as a mediator of haptic information between the ground and the participant's body. The haptic system, or touch feedback, works through the active exploration of the environment (static or dynamic), which involves the interpretation of spatiotemporal stimuli as they interact with several types of mechanoreceptors.^9^

The anchor system can help the vestibular system achieve a new adjustment of sensory mechanisms and/or improve the somatosensory system through the conflict reduction of information processing, which contributes to balance improvement. Therefore, this study aimed to evaluate the acute effects of the anchor system for balance evaluation in the upright position (limit of stability) on subjects with chronic dizziness of peripheral vestibular origin who have not responded to traditional VR.

## Methods

### Sample and setting

The sample was composed of subjects over 50 years of age of both sexes who presented with chronic dizziness and balance instability of peripheral vestibular origin as their main complaint.

These patients were admitted to Otorhinolaryngology Outpatient Clinic of the Department of Ophtalmology, Otorhinolaryngology and Head and Neck Surgery, School of Medicine, University of São Paulo (FMRP-USP), in the city of Ribeirão Preto, state of São Paulo, Brazil, where they were diagnosed by a doctor specializing in Otorhinolaryngology.

Patients with chronic dizziness of peripheral vestibular origin (*i.e.*, with symptoms that had occurred for at least three months after the first episode with no treatment)^10^ were referred to the Department of Speech Therapy, where they were treated with conventional VR. Patients who did not respond positively to rehabilitation for at least three months were referred to the Laboratory of Assessment and Rehabilitation of Equilibrium (LARE) to participate in this study.

The eligibility criteria included subjects of both sexes over 50 years of age diagnosed with chronic dizziness and decreased postural balance of peripheral vestibular origin unspecified dizziness or sensations of dizziness with peripheral etiology daily, weekly, and monthly episodes for at least six months; and symptoms of vertigo, dizziness, and lack of postural stability that did not respond positively to conventional VR, including the reorganization of the vestibulo-ocular reflex (VOR).

**Figure 1:**
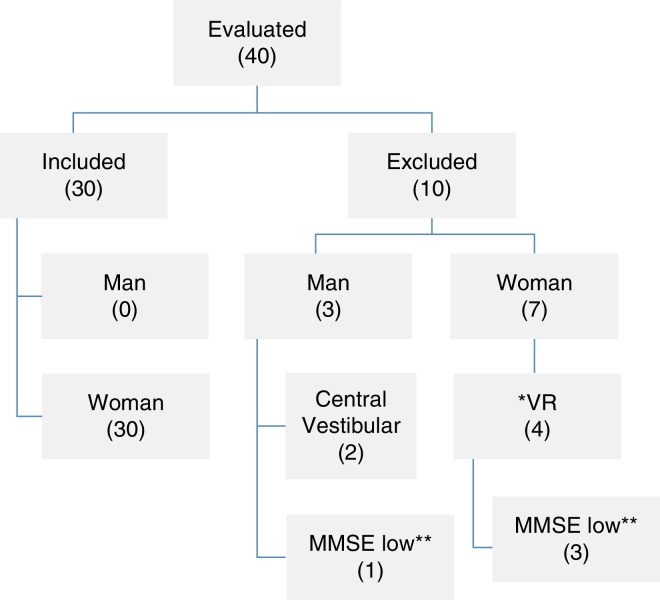
Flowchart of the sample (*VR, conventional vestibular rehabilitation in progress; **MMSE, Mini-Mental State Examination).

The exclusion criteria for the research included patients who received drugs (benzodiazepines and anticonvulsants) that affect balance or calcium channel blockers (cinnarizine and flunarizine) those who showed restricted mobility, visual restriction, and cognitive constraint that precluded the development of the assessments and intervention proposed or those who presented with systemic diseases without any drug control. Those who did not meet the eligibility criteria or did not agree to participate in the protocol received guidelines with regard to the importance of balance rehabilitation through physiotherapeutic care (Fig. 1).

The Mini-Mental State Examination (MMSE) was used to exclude those who presented with cognitive impairments.^3^, ^11^

This cross-sectional study was approved by the Ethics Committee on Human Research of the Ribeirão Preto Clinics Hospital Medicine School, University of São Paulo, Brazil, protocol N.3350/2013. All participants were informed about the study and agreed to participate by providing informed consent.

### Outcome measures

The balance assessment was performed during the limit of stability (LOS) test using the Balance Master^®^ system (Neurocom International, Inc., Clackamas, OR, United States).^12^, ^13^, ^14^

The LOS test is a dynamic test that analyzes an individual's ability to move the center of pressure (COP) in predetermined directions by asking the volunteer to transfer his weight as far as he can in eight pre-set directions around his central axis, using the ankle strategy instead of the hip, and without changing the support base. These targets are represented on the monitor, in real time, in front of the individual in eight points arranged in a circle. Each point is separated into angles of 45° and at a distance from the center that represents a difficulty level of 100% of the stability limit previously calculated by the machine, based on the individual's height. In this study, the points were established and represented by the numbers 1 (anterior), 3 (left lateral), 5 (posterior), and 7 (right lateral) to represent COP displacement in the anterior–posterior and medial–lateral directions.

To avoid inadequate postural strategies for the LOS test, which is not intuitive,^15^ all participants were allowed 5 min to become familiar with the platform and were given visual feedback and guidelines regarding strategies for COP displacement during the movements to be performed.

**Figure 2:**
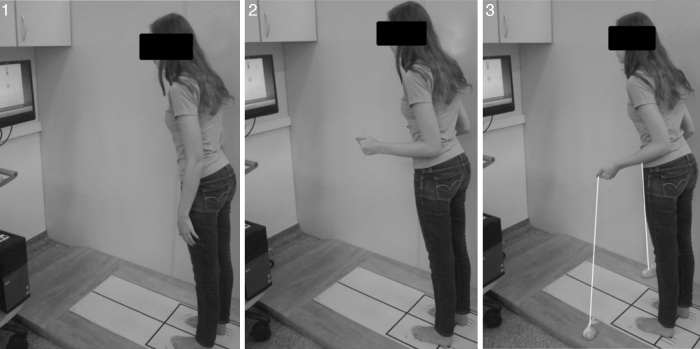
Positions adopted during evaluations using the Balance Master^®^ equipment. Position 1, standing with the arms along the body; position 2, standing with the elbows bent at 90° (simulating holding the anchors); and position 3, with elbows bent at 90° holding the anchors.

The individual was evaluated in three positions: position 1, standing with the arms along the body; position 2, standing with the elbows bent at 90° (simulating holding the anchors); and position 3, with the elbows bent at 90° holding the anchors (Fig. 2). Randomization was performed to determine the order of the positions. There was a five minute rest period between each position.

The anchor system is based on the development of a non-rigid tool, which consists of flexible ropes attached to a weight on the extremity that touches the ground; the volunteer holds the rope in the opposite extremity.^8^

In the position with anchors usage, the volunteer held the anchors to keep the flexible cables extended and the weight in constant contact with the ground.

The variables of the LOS assessed were^16^ movement latency (ML), average in seconds (s) from the visual stimulation to the beginning of the movement; endpoint excursion (EPE), defined as the higher COP displacement in the first sustained movement in each direction, measured in displacement percentage under the possible maximum displacement deemed to be 100%; and directional control of movement (DCM), defined as an individual's ability to maintain the axis of movement parallel to the targeted axis, measured as a percentage to compare the amount of intentional movement toward the target with the amount of corrective movement not directed toward the target. This is calculated following the formula (intentional movement − corrective movement/unintended movement) and is expressed as a percentage (%). If all voluntary movement is directed to the target in a straight line, the amount of corrective movement is equal to 0 and the score is 100%; the data obtained near this value are considered better.

With the aim of ensuring the safety of all assessments on the platform, the investigator in charge and an assistant stayed close to the participant to prevent any falling episodes and observed the movement strategies carried out by the volunteers during the tests.^17^

### Sample size calculation

To calculate the sample size, GraphPad StatMate software was used, and the variable of movement latency of the stability limit in the Balance Master^®^ system was considered a primary outcome, which resulted in sample size = 17, power = 0.9, and *α* error = 0.05.

### Statistical analysis

For the statistical analysis, the mean value of each variable after conducting three trials in each movement excursion was considered. All data were subjected to a descriptive analysis and normality test (Shapiro–Wilk) using SPSS^®^ software, v. 13.0. None of the data show normal distribution, so the statistical inference was performed using the nonparametric test (Kruskal–Wallis) to compare the different positions with and without the use of the anchor system. The level of significance was considered to be 95% based on Tukey's *post hoc* test.

## Results

Table 1 describes the anthropometric characteristics of the sample of 30 female volunteers. The male participants recruited did not meet the inclusion criteria of the study.

**Table 1:** Anthropometric characteristics of the evaluated sample.

Variables	Average ± SD (*n* = 30)
Age (years)	64.65 ± 8.69
Body mass (kg)	72.57 ± 7.66
Height (m)	1.61 ± 0.05
BMI (kg/m^2^)	28.14 ± 3.65

BMI, body mass index.

The following data describe the LOS test, in which the positions were selected to represent the anterior-posterior (1 and 5) and medial-lateral (3 and 7) oscillations.

For the ML variable, the anterior displacement was significantly lower with the use of the anchor system compared to the other positions evaluated (Table 2).

**Table 2:** Mean and standard deviation of the variable movement latency (ML) of the stability limit, in seconds.

Variables	Position 1	Position 2	Position 3	*p*-value	
				1 × 3	2 × 3
ML (1) seg	1.23 ± 0.40	1.64 ± 0.65	1.15 ± 0.47	0.003^a^	0.003^a^
ML (3) seg	1.23 ± 0.47	1.81 ± 0.98	1.51 ± 0.46	0.19	1.0
ML (5) seg	0.91 ± 0.55	1.30 ± 1.07	0.85 ± 0.51	1.0	0.57
ML (7) seg	1.23 ± 0.42	1.60 ± 0.77	1.30 ± 0.29	1.0	0.36

ML (1), anterior displacement; ML (3), right lateral displacement; ML (5), posterior displacement; ML (7), left lateral displacement. Position 1, standing with the arms along the body; position 2, standing with elbows bent at 90° (simulating holding the anchors); and position 3, elbows bent at 90° holding the anchors. 1 × 3, position 1 *vs.* position 3; 2 × 3, position 2 *vs.* position 3.

a Significant difference (*p* < 0.05) according to the Kruskal–Wallis test of multiple comparisons.

The point of maximum excursion (EPE) was greater when using the anchor than in position 1, which did not use the anchor and placed the arms along the body on the left lateral direction (Table 3).

**Table 3:** Mean and standard deviation of the variable endpoint excursion (EPE) of the stability limit, as a percentage.

Variables	Position 1	Position 2	Position 3	*p*-value	
				1 × 3	2 × 3
EPE (1) (%)	61.75 ± 19.10	58.93 ± 10.21	58.86 ± 22.49	1.0	1.0
EPE (3) (%)	67.70 ± 19.42	74.40 ± 18.22	71.98 ± 22.99	1.0	1.0
EPE (5) (%)	58.56 ± 21.79	60.13 ± 18.23	53.12 ± 13.42	1.0	0.72
EPE (7) (%)	59.21 ± 18.20	77.56 ± 18.11	73.94 ± 20.15	0.01^a^	1.0

EPE (1), anterior displacement; EPE (3), right lateral displacement; EPE (5), posterior displacement; EPE (7), left lateral displacement. Position 1, standing with the arms along the body; position 2, standing with the elbows bent at 90° (simulating holding the anchors); and position 3, elbows bent at 90° holding the anchors. 1 × 3, position 1 *vs.* position 3; 2 × 3, position 2 *vs.* position 3.

a Significant difference (*p* < 0.05) according to the Kruskal–Wallis test of multiple comparisons.

There was greater directional control (values closer to 100%) during the displacement in the anterior and posterior directions (*p* < 0.05) with the use of anchors compared to other positions, which means that the volunteer had greater motion control when directing toward the anterior and posterior targets after performing fewer corrective movements during the routine (Table 4).

**Table 4:** Mean and standard deviation of the variable directional control of movement (DCM) of the stability limit, as a percentage.

Variables	Position 1	Position 2	Position 3	*p*-value	
				1 × 3	2 × 3
DCM (1) (%)	84.02 ± 8.45	83.56 ± 5.33	88.85 ± 4.08	0.03^a^	1.0
DCM (3) (%)	86.63 ± 4.81	81.38 ± 4.61	80.15 ± 7.53	1.0	1.0
DCM (5) (%)	68.82 ± 22.81	63.34 ± 20.71	78.88 ± 7.99	0.001^a^	0.01^a^
DCM (7) (%)	78.21 ± 14.06	85.36 ± 3.81	87.12 ± 4.82	0.01	1.0

DCM (1), anterior displacement; DCM (3), right lateral displacement; DCM (5), posterior displacement; DCM (7), left lateral displacement. Position 1, standing with the arms along the body; position 2, standing with the elbows bent at 90° (simulating holding the anchors); and position 3, elbows bent at 90° holding the anchors. 1 × 3, position 1 *vs.* position 3; 2 × 3, position 2 *vs.* position 3.

a Significant difference (*p* < 0.05) according to the Kruskal–Wallis test of multiple comparisons.

## Discussion

The literature has shown that the better the postural balance, the better the functional capacity of subjects with chronic peripheral vestibular disorders; conversely the greater the impairment of functional capacity, the greater the risk of falls in these subjects.^16^

Peripheral vestibulopathy can create a chronic dysfunction condition in which activities performed in a given environment can cause conflicting sensory afferents, particularly when they require greater postural control that affects the stability limit of the individual.

The LOS has been evaluated to identify deficits of balance in individuals with chronic peripheral vestibulopathy.^16^ Studies have evaluated the LOS by computerized posturography to assess the functional stability of individuals with vestibular dysfunction, which enables the identification of possible changes in the elliptical area and oscillation speed of the pressure center.^18^, ^19^, ^20^

In 2010, a systematic review by Ricci et al., which was related to VR in middle-aged and elderly adults, found that in addition to the classic test evaluation of static and dynamic body balance, computed posturography was used in most studies, particularly in the LOS test of the latency of movement, maximum excursion point, and directional control of the movement variables.^20^

The mean age in the present study population was 64.6 years. According to published findings, some authors indicate age as a possible factor for the loss of vestibular function due to electrophysiological and structural changes in the vestibular system, which can have important consequences for postural control as early as 40 years of age and can cause multiple otoneurological symptoms, such as dizziness.^21^, ^22^

In addition, the prevalence of female subjects in the present study confirms what has already been described in the literature, that dizziness is more prevalent in women at a ratio of 2:1. An organic predisposition for vestibular disorders may be related to an intrinsic hormonal variation and/or metabolic disorders, and women suffer the most falls.^23^, ^24^, ^25^

The present study observed immediate benefits of using the anchor system in improving the LOS, particularly regarding the increase of the left lateral displacement with improved anterior and posterior directional control and lower anteriorly movement latency, according to the EPE, DCM, and ML variables, respectively.

Thus, the anchor system's function as a mediator of the haptic information between the ground and the user's body might be beneficial to improve balance performance.^9^, ^26^

Moreover, this system provides information about force, motion, texture, and shape (acceleration, gravitational, and inertial) involved in mechanical perception of the environment through the skin and kinesthetic system efforts, such as the touch of a finger with some static to reduce body oscillation.^26^

The benefits of the anchor system were also observed in the study of Freitas et al.^11^ in healthy elderly individuals who used the anchor system. Subjects were divided into three groups according to frequency of use of the anchoring system (0%, 50%, and 100%) in the standing position. The limited period of sessions at 50% improved postural control compared to the use of the anchor system for a longer time. The authors suggested that this result was due to the haptic information being used by the central nervous system as a way to recalibrate the process of sensory integration for improved postural control.^15^

Using the anchor system, the volunteers in the present study were able to move the COP more in left lateral displacement (EPE) compared to conditions without anchors and with the arm along the body.

Thus, the anchor system can be an important tool in rehabilitation of body balance programs, because a reduction in the stability limit in individuals with postural control impairments may cause difficulty when performing reaching tasks in the orthostatic position, such as picking up objects, or even when the individual is impacted by some external force.^16^ The smaller the stability limit, the worse is the ability of the individual to move without changing the support base, making him or her more susceptible to injury from falls.^17^

Regarding the ML, the subjects in this study took less time to start movement in the anterior direction using the anchor system compared to conditions without anchors and with the arm along the body. This can be related to the fact that when using the anchor system, the individual may feel a greater confidence to move toward the targets, using the haptic system as an adjunct to postural control system to keep the COP within the stability limit without changing the support base.

Another interesting finding of this study was the DCM variable, in which the use of the anchor system allowed the volunteer to reach the predetermined targets in the anterior and posterior displacement positions with more control, therefore performing the COP displacement with as little correction of movement as possible.

In 2011, the study by Izquierdo et al. subjected elderly patients with chronic peripheral vestibular disease to a rehabilitation program of body balance based on computerized dynamic posturography, noting an improvement of 13.8% in the DCM variable of the stability limit after two weeks of training.^18^ This was similar to the finding of the present study, in which the anchor system showed an immediate improvement of 10.06% in directional movement control.

The immediate positive effect obtained using the anchor system in subjects with vestibular disorder who did not show improvement after conventional VR suggests that it is a tool that can be used as a therapeutic strategy for balance improvement through recalibration of the sensory systems, mainly somatosensory.

As this is a relatively new way of exploring haptic information to improve postural control in individuals with chronic peripheral vestibulopathy, the physiological mechanism and ways in which the anchor system can improve postural control in these individuals have not been extensively explored in the literature.

However, if we compare the use of anchors with biofeedback modalities as a method of training for improved postural control, we can follow the theory of Horak, which suggests that sensory information provided frequently can be understood by the postural control system as an additional sensory system and may be used or further explored to the detriment of another system that is at a disadvantage or inaccurate.^25^

This study is relevant in demonstrating that the anchor system can improve the stability limit of the individual with chronic vestibular dysfunction, which can assist in posture control and can be used in exercise protocols for balance rehabilitation.

## Conclusion

The anchor system, by using haptic information for postural control, provided an immediate improvement in the stability limit in individuals with chronic peripheral vestibulopathy who had not responded to VR.

## Conflicts of interest

The authors declare no conflicts of interest.
